# Rapid and specific detection of porcine parvovirus using real-time PCR and High Resolution Melting (HRM) analysis

**DOI:** 10.1186/s12917-015-0364-2

**Published:** 2015-02-28

**Authors:** Hai-Qiong Yu, Xian-Quan Cai, Zhi-Xiong Lin, Xiang-Li Li, Qiao-Yun Yue, Rong Li, Xing-Quan Zhu

**Affiliations:** Technical Center, Guangdong Entry-Exit Inspection and Quarantine Bureau, Guangzhou, Guangdong Province 510630 PR China; Technical Center, Zhongshan Entry-Exit Inspection and Quarantine Bureau, Zhongshan, Guangdong Province 528403 PR China; Zhongshan Torch Polytechnic College, Zhongshan, Guangdong Province 528403 PR China; State Key Laboratory of Veterinary Etiological Biology, Lanzhou Veterinary Research Institute, Chinese Academy of Agricultural Sciences, Lanzhou, Gansu Province 730046 PR China

**Keywords:** Porcine parvovirus (PPV), High resolution melting (HRM), Real-time PCR

## Abstract

**Background:**

Porcine parvovirus (PPV) is the important causative agent for infectious infertility, which is a fairly tough virus that multiplies normally in the intestine of pigs without causing clinical signs in the world.

**Results:**

We developed an assay integrating real-time PCR and high resolution melting (HRM) analysis for the detection of PPV. Primers targeting the VP gene were highly specific, as evidenced by the negative amplification of closely related viruses, such as porcine circovirus 2 (PCV2), porcine reproductive and respiratory syndrome virus (PRRSV), pseudorabies virus (PRV), classical swine fever virus (CSFV), or Japanese encephalitis virus (JEV). The performance of unlabeled real time PCR was compared to TaqMan real time PCR, and the detection limits of the two methods were nearly equal. Moreover, there was good correlation between Cp and diluted genomic DNA when tested with the two methods. The assay has the accuracy of 100% in reference to labeled real time PCR, when it was tested on 45 clinical samples.

**Conclusions:**

The present study demonstrated that the established assay integrating real-time PCR and HRM is relatively cost-effective and more stable, which provides an alternative tool for rapid, simple, specific and sensitive detection of PPV.

## Background

Porcine parvovirus (PPV) is a major cause of reproductive failure in pigs. The PPV is a very resistant virus that can survive without a host for several months, which makes it difficult to remove the virus from the pig herd and its environment [1]. PPV does not cause clinical signs at any other time than during pregnancy, when it may cross the placenta and infect developing embryos and fetuses, resulting in resorption and mummification, abortion, stillbirth, neonatal death and reduced neonatal vitality [2]. PPV has been reported in many countries. Furthermore, although PPV has little if any effect on mature boars, they may act as non-infected carriers [3].

Several methods have been developed for rapid detection of PPV, including specific PCR, ELISA and LAMP [4-6]. Real-time PCR applications can be completed rapidly since no post-amplification modifications were required [7,8]. The analysis of amplified products by means of probes or melt curve analysis is furthermore highly accurate compared to analysis on agar gels. However, the probe-based real time PCR requires synthesis of the probe, which will increase the cost, and the probe is prone to degradation. Recently, high-resolution melting (HRM) for high-throughput analysis of many pathogens has been developed, for example, for variation scanning [9], genotyping [10] and species determination [11]. As different amplicons produce distinct melting curves, these can easily be compared to a reference melting curve to determine the identity of the amplicon [12]. Here, we developed a real-time PCR assay coupled with HRM analysis for the rapid and specific detection of PPV*.*

## Methods

### Ethics statement

This study was performed in strict accordance with the recommendations of the Animal Ethics Procedures and Guidelines of the People's Republic of China. The protocol was approved by the Animal Ethics Committee of Lanzhou Veterinary Research Institute, Chinese Academy of Agricultural Sciences (Permit No: LVRIAEC2013-010). Every effort was made to minimize suffering of the examined pigs.

### Genomic DNA extraction

A total of 45 pig tissue samples (liver, lung, spleen and kidney), suspected of being infected with PPV from different pig farms in Guangdong Province, China, were collected by The Center of Quality Test and Supervision for Breeding Swine (Guangzhou). The pigs suffered from a variety of clinical signs such as respiratory diseases, systemic diseases, diarrhea, or reproductive disorders. The ages of the pigs sampled ranged from neonatal to adult. One gram of the tissue was minced and diluted 1:10 in Dulbecco's Modified Eagle Medium (DMEM). Homogenization was carried out in a Stomacher 80. 60 μL extraction buffer and 10 μl proteinase K were added to 100 μl tissue homogenate supernatant, or cell culture inoculated with reference virus or closely related viruses. The mixture was incubated at 50°C for 2 h, DNA extractions were carried out with phenol/chloroform (1:1), refrigerated and centrifuged at 10000 rpm. Precipitation was performed with 95% ethanol for 18 h at -20°C and pellets were diluted in 25 μl sterile distilled water.

### Real time PCR detection of PPV with HRM analysis

Unlabelled real-time PCR amplification was performed in Lightcycler 480 (Roche, USA). A pair of primers designed targeting the VP1 gene, which is very important for PPV positioning within the host nucleus (sense: 5’-TCACCAAACAATTAATAATAGC-3’; PPV; antisense: 5’- GGTTCATCATCATTATATTGTG-3’) was synthesized by Takara Biotechnology (Dalian, China). The total reaction volume was 20 μl, consisted of 1 × HRM master mix (Roche, USA), 0.35 μM of each primer and 1 μl DNA as template. The PCR was carried out with initiation at 94°C for 10 min, followed by 10 cycles of 94°C, 30 s; annealing temperature step downs every 2 cycles of 1.0°C (from 63°C to 54°C). The annealing temperature for the final 25 cycles was 59°C with denaturation and extension phases as above. When PCR amplification was completed, HRM analysis was performed by lowering the temperature to 60°C for 5 min, followed by increasing the temperature ramping from 60°C to 95°C at 0.11°C/s, 25 acquisitions/°C. In this process, the PCR amplicons were allowed to denature and re-anneal in fluorescence with changes in temperature (dF/dT). The HRM profile was then analyzed using HRM analysis software, as reported previously [13].

### Real time PCR detection of PPV using TaqMan probe

The primers and probe were synthesized by Takara Biotechnology (Dalian, China) according to previous studies [7] targeting the VP2 gene as follows: (sense: 5-CCAAAAATGCAAACCCCAATA-3, antisense: 5-TCTGGCGGTGTTGGAGTTAAG-3), which amplified a fragment of 194 bp in length. The TaqMan probe, FAM- CTTGGAGCCGTGGAGCGAGCC-TAMRA, was used to detect any amplification. The real-time PCR amplification was carried out in a reaction mix of 20 μl TaqMan reaction mix consisted of 2.0 μl of 10× buffer, 0.4 μl of 10 mM dNTP, 3.6 μl of 25 mM MgCl_2_, 0.2 μl of 10 μM fluorogenic FAM-labeled PPV probe, 0.4 μl of 10 μM forward primer, 0.4 μl of 10 μM reverse primer, and 1 μl of DNA solution. The thermal conditions were as follows: one cycle at 94°C for 5 min; followed by 45 cycles at 94°C for 5 s, 60°C for 15 s. PCR amplification was performed by using the same thermal cycler LC 480 (Roche, USA).

## Results

### Specificity of the detection assay and confirmation of amplicon identity

The specificity of the primers was determined by performing PCR using pure genomic DNA from closely related viruses, including porcine circovirus 2 (PCV2), porcine reproductive and respiratory syndrome virus (PRRSV), pseudorabies virus (PRV), classical swine fever virus (CSFV), or Japanese encephalitis virus (JEV). No fluorescence signal was detected from all the ‘heterologous control samples’, as mentioned above. Moreover, A BLAST search of the chosen primers resulted in a hit of the target sequence in PPV*,* suggesting the specificity of the primers. To ensure the accuracy of the method, all amplicons were sequenced and proved highly homologous to corresponding sequences.

### Detection limit and correlation between Cp and diluted DNA

**Figure 1:**
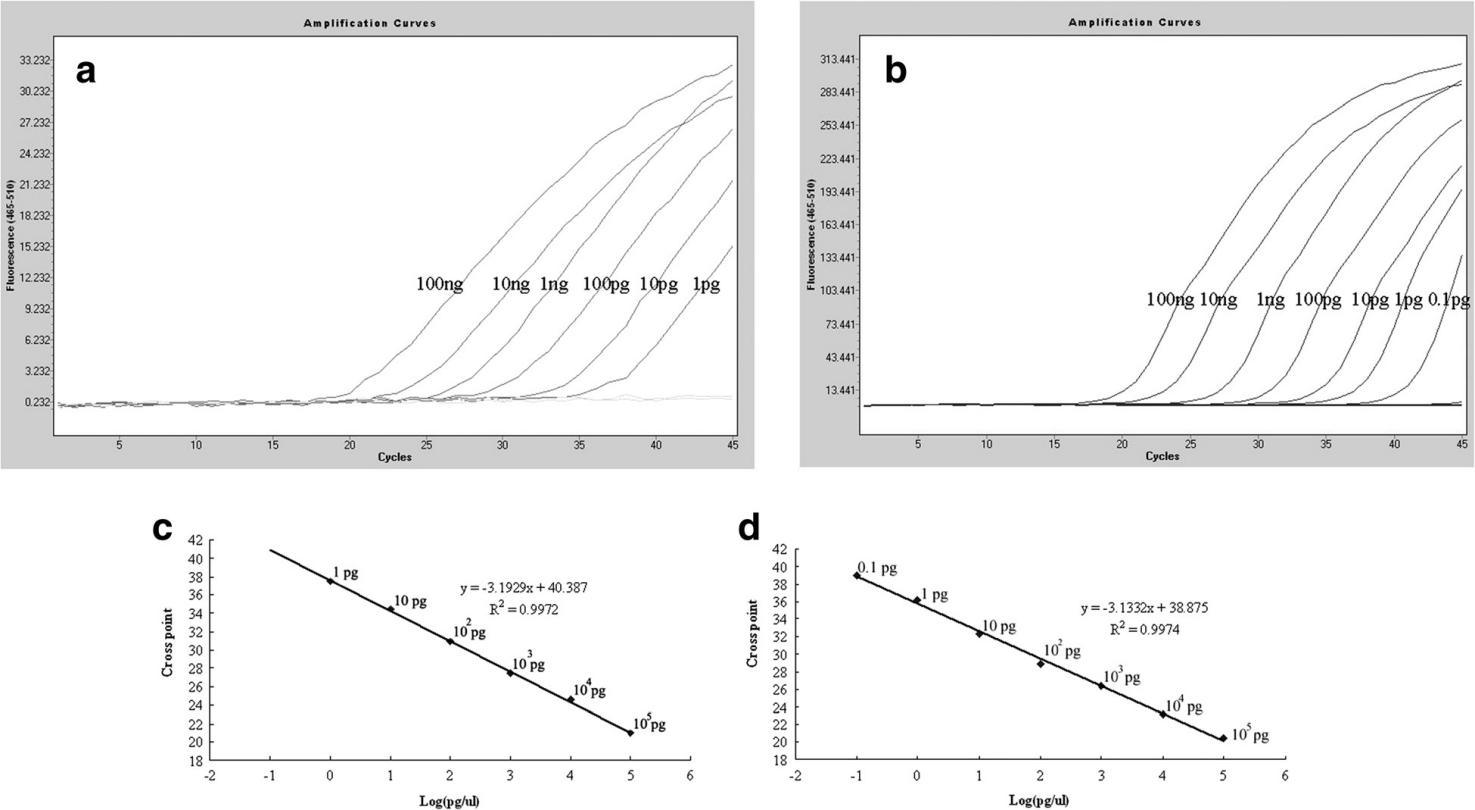
**Ten fold diluted genomic DNA of porcine parvovirus (PPV) tested with two kind of real time PCR (100 ng to 0.1 pg). (a)** TaqMan real time PCR amplification for 10 fold diluted genomic DNA; **(b)** Real time PCR with HRM analysis for 10 fold diluted genomic DNA; **(c)** A linear regression of the data providing a formula of y = -3.1929x + 40.387 (R^2^ = 0.9972) between Cp value and log concentration (100 ng to 1 pg), when performed with TaqMan real time PCR; **(d)** A linear regression of the data providing a formula of y = -3.1332x + 38.875 (R^2^ = 0.9974), between Cp value and log concentration (100 ng to 1 pg) when performed with real time PCR coupled with HRM analysis.

In order to evaluate the detection limit and correlation of the real-time PCR coupled with HRM analysis, the sensitivity of the real-time PCR was compared with a TaqMan real time PCR reported previously [7], serial dilutions of plasmid between 10^7^ copies and 10^1^ copies per reaction or 10-fold diluted genomic DNA was tested. Standard curves were generated using the protocol described above. The assay approaches the sensitivity of TaqMan real time PCR (Figure 1).

**Figure 2:**
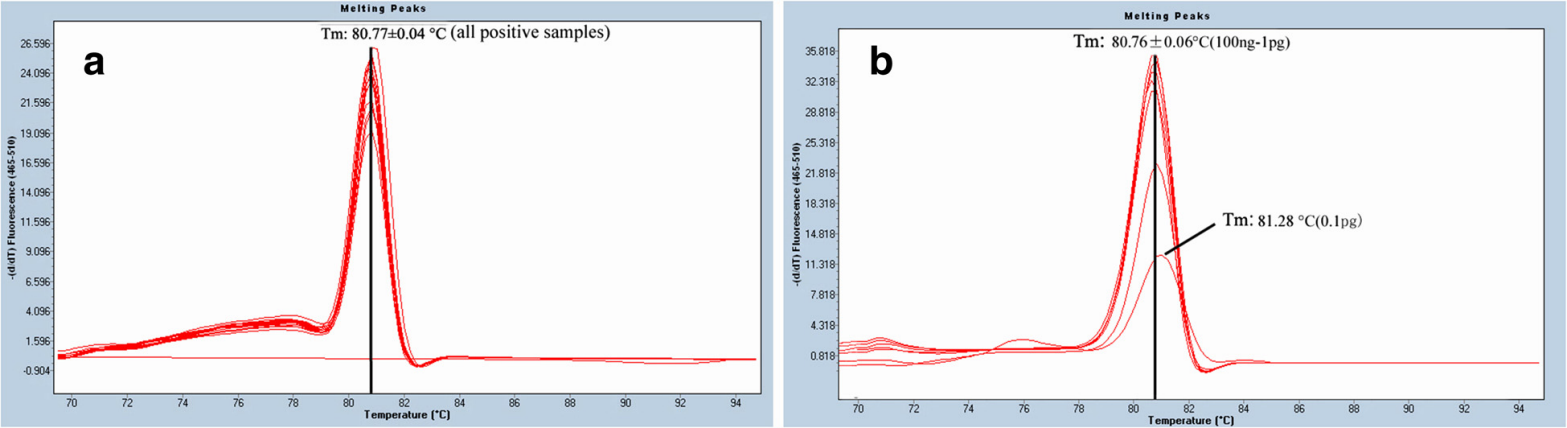
**Real time PCR and HRM analysis of serial diluted genomic DNA of porcine parvovirus (PPV). (a)** Melting peaks of amplicon from 12 positive field samples, 80.77 ± 0.04°C. **(b)** Melting peaks of amplicons of 10 fold diluted genomic DNA was 80.76 ± 0.06°C between 100 ng and 1 pg, while it is 81.28°C when the diluted DNA is 0.1 pg.

One pg genomic DNA and plasmid DNA of 10^1^ copies can be detected easily by both methods. The amplification curve evenly raised when applied with 0.1 pg viral DNA, but the melting curve shape of low amount DNA (0.1 pg) is obviously different with other dilutions (Figure 2b), and the Tm of 0.1 pg PPV DNA is 81.28°C while Tm of others dilutions is 80.76 ± 0.06°C. A good correlation was acquired. The formula between Cp value and concentration (100 ng to 1 pg) is y = -3.1929x + 40.387 (R^2^ = 0.9972), when performed with TaqMan real time PCR (Figure 1c), while it is y = -3.1332x + 38.875 (R^2^ = 0.9974), when performed with real time PCR coupled with HRM analysis (Figure 1d).

### Testing of clinical samples

Forty-five clinical pig samples were tested using both the real time PCR coupled with HRM analysis and TaqMan real time PCR. Results of the two assays were 100% consistent, and both methods showed that 12 of the examined samples were positive, while 33 were negative.

### HRM analysis

Constant HRM profiles with distinct Tm peaks were persistently obtained for all field samples and 10 diluted standard DNA. As shown in Figure 2, there is only one kind of characteristic profiles. The amplification products from all 12 positive samples had a Tm of 80.77 ± 0.04°C, and the 10 diluted standard DNA (100 ng - 1 pg) had a Tm of 80.76 ± 0.06°C. To ensure the accuracy of the method, the amplified products were sequenced. The results showed that all sequences were uniform with the HRM analysis results. The HRM analysis with different concentrations of the template appeared to be reliable, while the profile is unsatisfactory when the concentrations was 0.1 pg such as obviously different melting curve (Figure 2c), which is consistent with a previous report [13].

## Discussion

Diagnosis of PPV infection is very important for effective treatment and controlling the spread of infections. Here, we developed an unlabelled real-time PCR coupled with HRM assay targeting the VP1 gene which has been proven critical to replication efficiency in cell culture [14]. The data showed that there is a 26.7% positivity of all tested samples, which is lower than that of a previous study (41.6%) [5]. The reason may attribute to diverse regions and different sample sizes.

Melt curve analysis (MCA) is well known for identification of amplicons, but classical MCA can only distinguish gross differences (0.5°C) between PCR products [15], which will possibly make mistakes when tested with closely related viruses. HRM curve analysis allows for the detection of subtle sequence variations between products and provides a much more accurate comparison between amplicons [16]. Important to note is that this feature is mainly due to the binding characteristics of new generation dyes such as LC Green, which will saturates the molecule preventing dye relocation during the melting process. However, classical MCA was commonly applied with non-saturating dyes such as SYBR Green I.

To date, various detection and diagnostic techniques for detection of PPV have been developed [4-8]. There are, however, many drawbacks to these methods, for example, crude or recombinant antigens have been reported, however, the specificity and sensitivity of these methods are still in great need of improvement. TaqMan real-time PCR requires expensive probe, and LAMP has high risk of contamination.

Here, a touchdown PCR protocol was used that covers a range of annealing temperature between 63°C and 54°C. With touchdown PCR, a relatively high annealing temperature was used in the early cycles of PCR to ensure high accuracy of priming and amplification. Decreasing the annealing temperature in later cycles guarantees adequate amounts of PCR amplicons. With the help of saturates dyes such as LC green, the fluorescence of unlabelled real time PCR was nearly 10-fold stronger than TaqMan real time PCR (Figure 1b). It is well known that positive results should be proven by the obviously rising curve and exact temperature of melting (Tm) when tested with HRM. As shown in Figure 1, there is an ascending curve with 0.1 pg viral DNA, but the Tm of 0.1 pg PPV DNA is 81.28°C, while Tm of other dilutions is 80.76 ± 0.06°C. Hence, our results is not enough to prove that 0.1 pg PPV DNA can be accurately detected by HRM, so we could not conclude that it is more sensitive than TaqMan real time PCR. Less sensitivity of TaqMan real time assay was previously reported, because only single point mutation in TaqMan probe will decrease the sensitivity by 47% [17], other reasons for example, inefficiency of 5' exonuclease, and repeatedly thawing of probe may also contribute to it.

## Conclusions

The present study demonstrated that the real-time PCR platform coupled with HRM is an alternative tool for rapid, simple, specific and sensitive detection of PPV, which reduces turnaround time of the assay to almost 1 h, eliminates the risk of contamination, and saves expense. These features make it advantages for use in laboratories, and therefore could be a useful tool for determining the infected individuals.
